# Supplementary material for: Assessing Ethnic Minority Representation in Fibromyalgia Clinical Trials: A Systematic Review of Recruitment Demographics
Source: Int J Environ Res Public Health. 2023 Dec 15;20(24):7185. doi: 10.3390/ijerph20247185 (PMC10742509; doi:10.3390/ijerph20247185)
Supplement: Supplementary file 1 [file ijerph-20-07185-s001.zip › ijerph-2665559-supplementary.pdf]

Supplementary Table **S1**<br><br>Searches performed in databases to identify fibromyalgia clinical trials	
**Search performed<br>(All databases)**	**Filters applied<br>(Each database)**
Fibromyalgia **AND** "clinical trial* **OR** "randomised control trial" **OR** “randomized control trial”<br>**OR** RCT	**All databases**<br>2000 – June 2022<br><br>**PubMed**<br>Clinical Trial<br>Randomized Control Trial<br><br>**Web of Science**<br>Articles<br><br>**Cochrane Library**<br>Keyword in titles and abstracts<br><br>**Scopus**<br>Article<br>Language English

Supplementary Methods S1

Full inclusion and exclusion criteria

Type of article- Papers must be published as a primary research article published between the 1st of January 2000 and the 30th of June 2022, and printed in English.

Objectives and outcome- The aim of the studies must be the administration of a pharmaceutical agent(s) to either treat fibromyalgia symptoms, or to restore or measure the effect of drugs directly involved in neural transmission in areas of the nervous system involved in pain processing or the development other of of other fibromyalgia symptoms (e.g. neuroimaging studies that investigated changes to activity in the brain for this purpose were included). The aim of the studies had to be related to the treatment of fibromyalgia in all participants (i.e. studies investigating treatments for any other pain disorder in any of the participants were excluded). Studies testing the safety of drugs intended to treat fibromyalgia symptoms were also included (e.g. dose escalation studies). Exclusion criteria also applied to studies investigating optimal methodologies or statistical analyses in clinical trials, surveys measuring patterns of drug use in fibromyalgia, and studies that used pharmaceuticals to induce pain to study fibromyalgia pain modulation. All studies recruited new participants (i.e. they were not continuation studies or post hoc analyses of previous studies), except for two selected studies for Objective 4.

Type of treatment in studies- To meet the inclusion criteria, studies had to state the active pharmacological agent(s) being administered to the participants. Studies where multiple combinations of pharmaceutical agents were used together or where the combinations of medications were not specified were excluded (e.g. homeopathy). Pharmaceuticals could have been taken in any form, e.g. tablet, topical, liquid injected, or inhaled. Studies must have been investigating pharmaceutical agents only, i.e., exclusion criteria applied to studies comparing non-pharmacological treatments with pharmacological treatments and/or investigating pharmacological treatments in combination with non-pharmacological treatments.
